# Characteristics and mortality rates among patients requiring intermediate care: a national cohort study using linked databases

**DOI:** 10.1186/s12916-021-01912-x

**Published:** 2021-02-12

**Authors:** Catherine J. Evans, Laura Potts, Ursula Dalrymple, Andrew Pring, Julia Verne, Irene J. Higginson, Wei Gao, on behalf of SPACE, Sube Banerjee, Marsha Dawkins, Clare Ellis-Smith, Claire Goodman, Christine Norton, Mathew Maddocks, David Seamark

**Affiliations:** 1 King’s College London, Cicely Saunders Institute of Palliative Care, Policy and Rehabilitation, Bessemer Road, London, SE5 9PJ England; 2 Sussex Community NHS Foundation Trust HQ, Brighton General Hospital, Elm Grove, Brighton, BN2 3EW England; 3 Public Health England, 2 Rivergate, Redcliffe, Bristol, BS1 6EH England

**Keywords:** Intermediate care facilities, Health services for the aged, Subacute care, Geriatrics, Palliative care, Mortality, Cohort studies

## Abstract

**Background:**

Adults increasingly live and die with chronic progressive conditions into advanced age. Many live with multimorbidity and an uncertain illness trajectory with points of marked decline, loss of function and increased risk of end of life. Intermediate care units support mainly older adults in transition between hospital and home to regain function and anticipate and plan for end of life. This study examined the patient characteristics and the factors associated with mortality over 1 year post-admission to an intermediate care unit to inform priorities for care.

**Methods:**

A national cohort study of adults admitted to intermediate care units in England using linked individual-level Hospital Episode Statistics and death registration data. The main outcome was mortality within 1 year from admission. The cohort was examined as two groups with significant differences in mortality between main diagnosis of a non-cancer condition and cancer. Data analysis used Kaplan-Meier curves to explore mortality differences between the groups and a time-dependant Cox proportional hazards model to determine mortality risk factors.

**Results:**

The cohort comprised 76,704 adults with median age 81 years (IQR 70–88) admitted to 220 intermediate care units over 1 year in 2016. Overall, 28.0% died within 1 year post-admission. Mortality varied by the main diagnosis of cancer (total *n* = 3680, 70.8% died) and non-cancer condition (total *n* = 73,024, 25.8% died). Illness-related factors had the highest adjusted hazard ratios [aHRs]. At 0–28 days post-admission, risks were highest for non-cancer respiratory conditions (pneumonia (aHR 6.17 [95%CI 4.90–7.76]), chronic obstructive pulmonary disease (aHR 5.01 [95% CI 3.78–6.62]), dementia (aHR 5.07 [95% CI 3.80–6.77]) and liver disease (aHR 9.75 [95% CI 6.50–14.6]) compared with musculoskeletal disorders. In cancer, lung cancer showed largest risk (aHR 1.20 [95%CI 1.04–1.39]) compared with cancer ‘other’. Risks increased with high multimorbidity for non-cancer (aHR 2.57 [95% CI 2.36–2.79]) and cancer (aHR 2.59 [95% CI 2.13–3.15]) (reference: lowest).

**Conclusions:**

One in four patients died within 1 year. Indicators for palliative care assessment are respiratory conditions, dementia, liver disease, cancer and rising multimorbidity. The traditional emphasis on rehabilitation and recovery in intermediate care units has changed with an ageing population and the need for greater integration of palliative care.

**Supplementary Information:**

The online version contains supplementary material available at 10.1186/s12916-021-01912-x.

## Background

Adults increasingly live and die with chronic progressive conditions into advanced age. The *Lancet Commission* in 2017 identified living with chronic illness that compromises physical, social or emotional function as a construct of serious health-related suffering relieved by palliative care where quality of life is the main goal of care [1]. Globally, the greatest increase in serious health-related suffering is projected for people aged 70 years and over [2]. Living into advanced age is often accompanied by multimorbidity and frailty and an uncertain illness trajectory of gradual decline over many years into end of life [3–5]. This trajectory is punctuated by points of marked decline from an often seemingly minor event, like an infection and risk of poor outcomes including hospitalisation and death [4]. Unplanned hospitalisations are common and rise with nearness of end of life [6, 7]. This trajectory is well described, but less understood are the priorities for high-quality care for people to live as well as possible with advancing age and chronic conditions [5].

Inpatient intermediate care units, such as a community hospital or post-acute facility, are an important part of the continuum of care to manage the care needs for people with chronic progressive conditions. They care increasingly for a mainly older population to provide time-limited transitional care between different levels of care and settings, for example, acute hospital and home. Care focuses on supporting recovery and function following points of decline with emphasis on comprehensive geriatric assessment, enablement and rehabilitation [8, 9]. These units are generally small (≤ 30 beds) [10] and provide subacute services tailored to the needs of the local population with varying access to specialist services such as a geriatrician, alongside the core staff [8, 9]. Patients and family carers describe these facilities positively placing particular value on location close to home that enables holistic and personalised care facilitated by multi-disciplinary team working and support for difficult psychological transitions, for example, loss of independence with disease progression [10]. Systematic review evidence demonstrates that compared with acute hospital care, patients and family carers in intermediate care units report better experiences and care is cost-effective to support post-acute recovery and rehabilitation [8, 11]. However, less considered is risk of end of life and the need for care orientated towards quality of life and comprehensive palliative care assessment.

Orientating services to changing population needs and identifying priorities to ensure high-quality care is best informed by understanding care needs at a population-level. However, there is a dearth of population-based evidence on the characteristics of adults admitted to intermediate care units and nearness of death and the associated factors. Our review of evidence before this study on mortality over 1 year post-admission to an intermediate care unit (community hospital, post-acute care or skilled nursing facility) identified twelve studies [12–23] reporting approximately 661,751 participants (Additional file D: Tables D1-D2). No studies considered the total population at the national level or indicators for care where quality of life is the main goal. Rather, the studies reported discrete populations by a specific disease(s), intervention or region to identify factors associated with mortality, for example, physical function and quality of care. This study aimed to examine the patient cohort characteristics and factors associated with mortality over 1 year post-admission to an intermediate care unit in England. The findings intended to inform policy and clinical priorities to deliver high-quality care for people in transition between hospital and home and requiring intermediate care.

## Methods

### Study design, data sources and participants

A national retrospective cohort study using linked databases. These included National Health Service (NHS) Hospital Episode Statistics (HES) linked at the individual-level to the Office for National Statistics (ONS) death registration data. Intermediate care units were defined in the HES data as a community hospital with inpatient beds in England. Data were extracted for all adults admitted to an intermediate care unit, 01/01/2016 to 31/12/2016 as the most complete datasets. NHS Digital supplied the HES data and ONS death registration records data linked by an individual anonymised identifier to enable deterministic linkage [24]. HES data details information about all patients admitted to NHS hospitals in England [25]. It captures illnesses and related conditions, with each electronic record containing up to 20 diagnosis fields coded according to International Classification of Diseases, 10th edition (ICD-10) [26]. The population cohort included adults (≥ 18 years) admitted for ≥ 1 nights to an eligible intermediate care unit between 01/01/2016 and 31/12/2016, with 12-month follow-up until end of 2017. Individuals were excluded using outpatient (e.g. day case) or maternity facilities. Reporting follows the STROBE guideline [27] and the RECORD [28] extension for routine data (Additional file A: Table A1).

### Procedures

The vital status (alive or dead) of the cohort was identified from the linked mortality data for date of death over the 12 months from the first admission (the index admission) to an intermediate care unit. Patients where a linked death record was not identified were assumed to have survived. HES data on hospital admission were aggregated into a patient “spell” in a single hospital. Each spell encompassed all episodes of care reported in the identified intermediate care unit to confirm the admission and discharge dates. Discharge included to usual residence, transfer to another medical facility or death.

All intermediate care units in England in the HES data were reviewed to identify those with inpatient facilities and reported admissions of ≥ 1 night in 2016. But identifying the facilities was convoluted. These facilities are not automatically differentiated in the HES database. Initially, facilities were identified from the names and postcodes from the Community Hospital Association (CHA), UK database [29] and each postcode checked with the respective facility website. Each confirmed postcode was mapped to the NHS site code in the NHS Trust Site database [30] and each site code then checked in the NHS successor archive database [31] to remove facilities that closed in 2016.

### Main outcome and covariates

The main outcome was mortality within 1 year from the index admission. The last year of life is considered a key indicator for likely benefit from palliative care [32]. The covariates examined as associated with end of life included demographic, illness and environmental factors. Demographic covariates included age (grouped for clinical relevance), sex and ethnicity. Illness factors were the main diagnosis derived from the first recorded diagnosis code on the index admission. Codes were recorded in ICD-10 and grouped using respective chapter codes (Additional File C: Tables C4-C5). Comorbidities were calculated from the Charlson comorbidity index for each person using the R ‘comorbidity’ package (version 0.5.3.9) [33, 34]. All recorded diagnoses were collated from the index admission and hospital episodes over 1 year before the index admission [35]. Environment covariates included the admission setting (e.g. post-acute), admission type categorised as elective when the decision to treat was prescribed before the admission, or non-elective, and level of deprivation by usual place of residence using index of multiple deprivation indices [36] at Lower Super Output Area (LSOA).

### Statistical analysis

Patient socio-demographics and clinical characteristics were described with descriptive statistics. Kaplan-Meier curves were used to visualise survival probabilities by respective explanatory variables. The multivariate survival analysis used a time-dependant Cox proportional hazards (PH) model to determine factors associated with mortality. All descriptive socio-demographic and clinical variables (see Table 1) were examined for inclusion in the survival model. The model included only complete cases for the identified covariates.

**Table 1:** Demographics of the study population

	Non-cancer	Cancer	All
**Total number of patients**	73,024	3680	76,704
**Mortality 365 days from admission**	18,840 (25.8%)	2605 (70.8%)	21,477 (28.0%)
**Days until death from admission**			
0–28 nights	3615 (19.2%)	1433 (55.0%)	5048 (23.5%)
29–180 nights	9587 (50.8%)	964 (37.0%)	10,551 (49.2%)
181–365 nights	5657 (30.0%)	209 (8.0%)	5866 (27.3%)
**Age (years)**			
Mean (SD)	76.8 (15.9)	73.9 (12.8)	76.7 (15.8)
Median (IQR)	81 (71–88)	76 (67–83)	81 (70–88)
**Age (years)**			
18–39	3226 (4.4%)	60 (1.6%)	3286 (4.3%)
40–64	9103 (12.5%)	685 (18.6%)	9788 (12.8%)
65–74	10,927 (15.0%)	976 (26.5%)	11,903 (15.5%)
75–84	21,848 (29.9%)	1194 (32.5%)	23,042 (30.0%)
85–94	24,657 (33.8%)	713 (19.4%)	25,370 (33.1%)
95+	3263 (4.5%)	52 (1.4%)	3315 (4.3%)
**Sex**			
Female	43,228 (59.2%)	1932 (52.5%)	45,100 (58.8%)
Male	29,793 (40.8%)	1748 (47.5%)	31,601 (41.2%)
**Ethnicity**			
White	69,892 (95.7%)	3574 (97.1%)	73,466 (95.8%)
Black and ethnic minority	1930 (2.6%)	41 (1.1%)	1971 (2.6%)
Unknown	1202 (1.7%)	65 (1.8%)	1267 (1.7%)
**Charlson comorbidity index**			
Median (IQR)	1 (0–3)	8 (3–9)	2 (0–3)
Range	0–18	2–17	0–18
**Charlson comorbidity index**			
0	20,155 (27.6%)	0 (0.0%)	20,155 (26.3%)
1–2	28,386 (38.9%)	713 (19.4%)	29,099 (37.9%)
3–4	15,275 (20.9%)	667 (18.1%)	15,942 (20.8%)
≥ 5	9208 (12.6%)	2300 (62.5%)	11,508 (15.0%)
**Admission type**			
Elective	16,535 (22.7%)	1257 (34.2%)	17,828 (23.3%)
Non-elective	56,307 (77.3%)	2418 (65.8%)	58,689 (76.7%)
**Admitted from**			
Hospital (ED or general hospital)	33,553 (46.0%)	1360 (37.0%)	34,913 (45.5%)
Home (personal dwelling/care home)	36,585 (50.1%)	2286 (62.1%)	38,871 (50.7%)
Other	617 (0.8%)	15 (0.4%)	632 (0.8%)
Unknown	2269 (3.1%)	19 (0.5%)	2288 (3.0%)
**Length of stay in an intermediate care unit**			
Median (IQR)	17 (5–34)	8 (5–34)	17 (5–34)
Range	0–1073	0–190	0–1073
**Length of stay in an intermediate care unit**			
0–25 nights	46,916 (64.6%)	2961 (80.6%)	49,877 (65.4%)
26+ nights	25,733 (35.4%)	712 (19.4%)	26,445 (34.7%)

Data are n (%) and represent status at index admission to the intermediate care unit. Charlson Index includes all malignancy, including lymphoma and leukaemia, except malignant neoplasm of skin [35]. Abbreviations: *SD* standard deviation, *IQR* interquartile range, *ED* emergency department. Definition: home and community setting, e.g. own home, care home (with or without nursing) but not residential accommodation where medical care is provided, e.g. inpatient hospice

The modelling procedure for the multivariate analysis was as follows. First, data were split into two cohorts, of main diagnosis non-cancer condition or cancer at the index admission. Sensitivity analysis showed that the survival profile for a main diagnosis of a non-cancer condition was significantly different from cancer. Individuals with a main diagnosis of a non-cancer condition may have had a history of cancer, but this was not reported as the reason for admission. Two separate models were fitted to accommodate the difference in mortality between the non-cancer and cancer groups. Initially, a stepwise variable selection procedure obtained the best Cox PH model for each cohort, using bidirectional selection with *p* value entry criterion 0.1 and retention criterion of 0.05 (Additional file B). The models were selected that had the maximum concordance statistic (a measure of goodness of fit) and contained the fewest covariates, retaining those with clinical importance (e.g. admission type). The Schoenfeld residuals of each covariate were tested for proportional hazards. However, the non-proportional hazards violated the assumptions of the Cox PH approach. To address this, time-dependant coefficients were used for covariates that had a non-stationary effect on mortality overtime (e.g. main diagnosis) [37]. This meant that coefficients for covariates that violated the proportional hazards assumption could vary as a step function overtime [37]. Time intervals of 28 days in the cancer model and 28 days and 180 days in the non-cancer model were identified by depicting the original hazard estimate against the time-varying estimate of a given covariate. The final model fit was assessed by visually comparing observed to expected hazard plots and observing concordance statistics. All analyses were completed in R (version 3.6.1). All statistical tests were 2-sided at a level of *p* ≤ 0.05.

### Research ethics statement

The study used anonymous national datasets. ONS Individual Approvals for full data access were held by LP, UD and AP. CJE and WG accessed the aggregated data. Ethical approval was not required.

## Results

### Study population

**Fig. 1:**
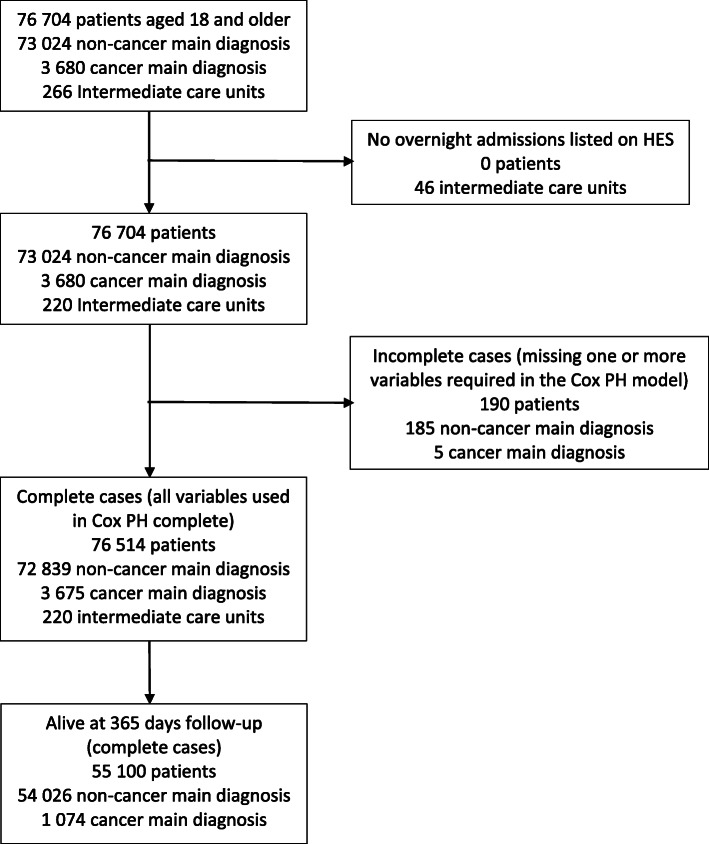
Study cohort profile

From 01/01/2016 to 31/12/2016, 76,704 individual adults were admitted to 220 intermediate care units in England (Fig. 1). The facilities were in both urban and rural localities. Most facilities had a single ward (71%) providing general medical care (Additional file C: Tables C2-C3). We identified 266 intermediate care units but excluded 46 (17.3%) with no admissions recorded, because of, for example, closure of inpatient beds.

Table 1 shows the characteristics of the cohort. Most patients were older with a median age at admission of 81 years (IQR 70–88, range 18–107), ethnically white (95.8%) and female (58.8%). The population formed two overarching groups identified by the main admission diagnosis of cancer (*n* = 3680, 4.8%) or non-cancer condition (*n* = 73,024, 95.2%) with significant difference in mortality (70.8% vs. 25.8% respectively). The main cancer site were digestive organs (23.6%) (Additional file C: Tables C1, C4-C5). The non-cancer conditions were highly heterogeneous with prominent conditions of injuries (17.0%) and ‘unknown and unspecified causes of morbidity’ (12.7%) (Additional file C: Table C1, C4-C5). Multimorbidity was common (73.7%) and highest in the cancer group (62.5% highest score of ≥ 5). Admission were non-elective (total *n* = 58,689, 76.7%) from hospital (45.5%) or usual residence (50.7%).

### Main outcome

**Fig. 2:**
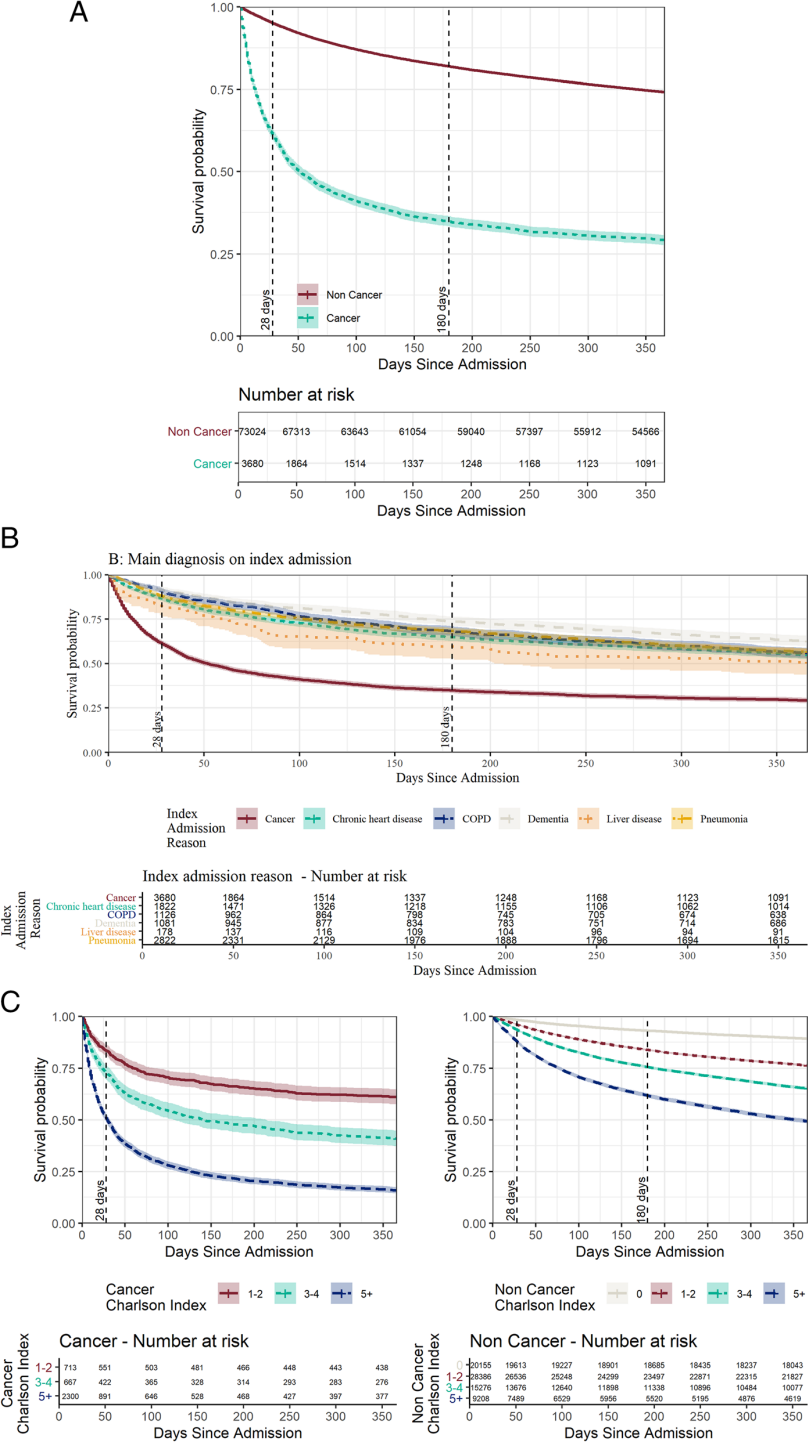
Kaplan-Meier survival curves and 95% confidence intervals by **a** main admission reason relating to cancer and non-cancer, **b** main diagnosis and **c** Charlson comorbidity index by cancer and non-cancer

Mortality over 1 year varied by diagnosis (cancer 2605, 70.8%), and non-cancer (18,840, 25.8% cases died) (see Fig. 2a). Deaths in the cancer group largely occurred 0 to 28 days post-admission (1433, 55.0%), and in the non-cancer group 29 to 180 days post-admission (9587, 50.8%) (Fig. 2a, Table 1). In the non-cancer group, chronic heart disease, respiratory diseases (pneumonia and chronic obstructive pulmonary disease [COPD]), dementia and liver disease had lowest survival probability over time (see Fig. 2b). Increasing comorbidities showed declining survival probability in both the cancer and non-cancer groups (see Fig. 2c). In both groups, the index admission was mainly non-elective (non-cancer 56,307, 77.3%; cancer 2418, 65.8% cases), with similar proportions admitted post-acute (non-cancer 33,553, 46.0%; cancer 1360, 37.0%) or usual residence (non-cancer 36,585, 50.1%; cancer 2286, 62.1%). Survival increased with decreasing age in both groups (Additional file C: Fig.C1, Kaplan-Meir curve by age categories).

The multivariate analysis used two models of non-cancer (72839) and cancer (3675), total 76,514 complete cases. Tables 2 and 3 show the respective model and the factors associated with mortality. The models included six covariates comprising age, sex, comorbidities (Charlson index score), main diagnosis, admission type and admission setting. To meet the hazards assumption of variation by time, step functions were employed at 28 days and 29–365 days in the cancer model, and 0–28 days, 29–180 and 181–365 days in the non-cancer model.

**Table 2:** Non-cancer model and factors associated with mortality within 1 year post-admission (complete cases *n* = 72,839)

Covariate [*denotes reference group]	0 to 365 days	Hazard up to 28 days^**a**^		Hazard 29 to 180 days^**b**^		Hazard 181 to 365 days^**c**^	
	*n*/*N* (%)	*n*/*N* (%)	Adjusted hazard ratio (95% CI)	*n*/*N* (%)	Adjusted hazard ratio (95% CI)	*n*/*N* (%)	Adjusted hazard ratio (95% CI)
**Age (years)**							
18–64	683/12,288 (6%)	191/12,288 (2%)	**0.38 (0.32, 0.44)**	313/12,097 (3%)	**0.17 (0.15, 0.19)**	179/11,784 (2%)	**0.14 (0.12, 0.16)**
65–74	1885/10,894 (17%)	407/10,894 (4%)	**0.58 (0.52, 0.65)**	906/10,487 (9%)	**0.41 (0.38, 0.44)**	572/9581 (6%)	**0.41 (0.38, 0.45)**
75–84	5731/21,796 (26%)	1101/21,796 (5%)	**0.7 (0.65, 0.75)**	2852/20,695 (14%)	**0.6 (0.57, 0.62)**	1778/17,843 (10%)	**0.62 (0.59, 0.66)**
85–94*	10,514/27,861 (38%)	1911/27,861 (7%)	1	5 00/25,950 (21%)	1	3103/20,450 (15%)	1
**Sex**							
Male*	8779/29,759 (30%)	1789/29,759 (6%)	1	4476/27,970 (16%)	1	2514/23,494 (11%)	1
Female	10,034/43,080 (23%)	821/43,080 (4%)	**0.8 (0.75, 0.85)**	5095/41,259 (12%)	**0.76 (0.73, 0.80)**	3118/36,164 (9%)	**0.76 (0.72, 0.80)**
C**harlson comorbidity index**							
0	2172/20,088 (11%)	341/20,088 (2%)	**0.59 (0.52, 0.67)**	1050/19,747 (5%)	**0.58 (0.54, 0.63)**	781/18,697 (4%)	**0.66 (0.60, 0.71)**
1–2*	6702/28,320 (24%)	1158/28,320 (4%)	1	3398/27,162 (13%)	1	2146/23,764 (9%)	1
3–4	5290/15,249 (35%)	990/15,249 (6%)	**1.39 (1.28, 1.52)**	2737/14,259 (19%)	**1.41 (1.34, 1.48)**	1563/11,522 (14%)	**1.36 (1.28, 1.45)**
5^+^	449/9182 (51%)	1121/9182 (12%)	**2.57 (2.36, 2.79)**	2386/8061 (30%)	**2.31 (2.19, 2.44)**	1142/5675 (20%)	**2.06 (1.92, 2.22)**
**Admission type**							
Non-elective*	16,646/56,270 (30%)	3169/56,270 (6%)	1	8490/53,101 (16%)	1	4987/44,611 (11%)	1
Elective	2167/16,569 (13%)	441/16,569 (3%)	**0.81 (0.73, 0.90)**	1081/16,128 (7%)	**0.72 (0.67, 0.77)**	645/15,047 (4%)	**0.67 (0.62, 0.73)**
**Setting admitted from**							
Home* (personal dwelling/care home)	8005/36,442 (22%)	1421/36,442 (4%)	1	4233/35,021 (12%)	1	2351/30,788 (8%)	1
Hospital	10,115/33,529 (30%)	2092/33,529 (6%)	**1.26 (1.18, 1.35)**	4987/31,437 (16%)	0.97 (0.93, 1.01)	3036/26,450 (11%)	**1.07 (1.02, 1.13)**
Other and unknown	693/2868 (24%)	97/2868 (3%)	**0.74 (0.60, 0.91)**	351/2771 (13%)	**0.83 (0.75, 0.93)**	245/2420 (10%)	1.02 (0.89, 1.16)
**Main diagnosis on admission (ICD-10 code)**							
Musculoskeletal disorders* (M)	855/9229 (9%)	96/9229 (1%)	1	450/9133 (5%)	1	309/8683 (4%)	1
Injury (S, T)	2770/13,002 (21%)	299/13,002 (2%)	**1.38 (1.09, 1.74)**	1495/12,703 (12%)	**1.4 (1.26, 1.56)**	976/11,208 (9%)	**1.35 (1.18, 1.53)**
Renal and genitourinary disease (N)	1478/4820 (31%)	257/4820 (5%)	**3.05 (2.41, 3.87)**	783/4563 (17%)	**2 (1.78, 2.25)**	438/3780 (12%)	**1.86 (1.60, 2.16)**
Cerebrovascular disease (I60–68)	769/3067 (25%)	191/3067 (6%)	**3.02 (2.35, 3.88)**	375/2876 (13%)	**1.36 (1.19, 1.57)**	203/2501 (8%)	1.12 (0.94, 1.35)
Digestive diseases (K)	613/2828 (22%)	133/2828 (5%)	**3.47 (2.67, 4.51)**	306/2695 (11%)	**1.77 (1.53, 2.05)**	174/2389 (7%)	**1.57 (1.31, 1.90)**
Pneumonia (J)	1223/2821 (43%)	341/2821 (12%)	**6.17 (4.90, 7.76)**	553/2480 (22%)	**2.31 (2.03, 2.62)**	329/1927 (17%)	**2.34 (2.00, 2.74)**
Chronic heart disease (I)	823/1819 (45%)	242/1819 (13%)	**6.14 (4.83, 7.81)**	399/1577 (25%)	**2.41 (2.10, 2.76)**	182/1178 (15%)	**1.89 (1.57, 2.28)**
Infections (A, B)	485/1411 (34%)	96/1411 (7%)	**3.69 (2.78, 4.91)**	274/1315 (21%)	**2.41 (2.07, 2.81)**	115/1041 (11%)	**1.67 (1.34, 2.07)**
COPD (J40–44)	496/1125 (44%)	105/1125 (9%)	**5.01 (3.78, 6.62)**	252/1020 (25%)	**2.86 (2.45, 3.34)**	139/768 (18%)	**2.76 (2.26, 3.39)**
Dementia (F00–03, G30–31)	396/1046 (38%)	92/1046 (9%)	**5.07 (3.80, 6.77)**	187/954 (20%)	**2.17 (1.82, 2.57)**	117/767 (15%)	**2.23 (1.80, 2.76)**
Acute heart disease (I)	304/955 (32%)	62/955 (6%)	**3.38 (2.45, 4.66)**	175/893 (20%)	**2.13 (1.78, 2.54)**	67/718 (9%)	**1.32 (1.01, 1.73)**
Blood diseases (D)	223/574 (39%)	45/574 (8%)	**4.47 (3.13, 6.38)**	118/529 (22%)	**2.66 (2.17, 3.26)**	60/411 (15%)	**2.49 (1.89, 3.29)**
Liver disease (B17–B18, C22, K70–75)	88/178 (49%)	32/178 (18%)	**9.75 (6.50, 14.6)**	40/146 (27%)	**4.06 (2.93, 5.62)**	16/106 (15%)	**3.23 (1.95, 5.36)**
Mental/behavioural disorders (F)	240/2418 (10%)	28/2418 (1%)	1.37 (0.89, 2.09)	124/2390 (5%)	**1.42 (1.16, 1.73)**	88/2266 (4%)	**1.51 (1.19, 1.92)**
Other	8050/27,546 (29%)	1591/27,546 (6%)	**3.6 (2.92, 4.43)**	4040/25,955 (16%)	**2.01 (1.82, 2.22)**	2419/21,915 (11%)	**1.88 (1.67, 2.12)**

Data in bold indicates significant findings. Data are *n* (%) and represent status at index admission to the intermediate care unit. Adjusted hazard ratio (95% CI). Main diagnosis on admission (ICD-10 chapter code), full codes Additional File Tables C1 and C4. ^a^Hazard of death in the first 28 days of admission. ^b^Hazard of death within 29 to 180 days of admission, which excludes patients that died before 28 days. ^c^Hazard of death within 181 to 365 days of admission, which excludes patients that died before 180 days. Abbreviations: *ICD-10* International Classification of Diseases 2010, *COPD* chronic obstructive pulmonary disease

**Table 3:** Cancer model and factors associated with mortality within 1 year post-admission (complete cases *n* = 3675)

	0 to 365 days	Hazard up to 28 days^**a**^		Hazard 29 to 365 days^**b**^	
Covariate reference group*	*n*/*N* (%)	*n*/*N* (%)	Adjusted hazard ratio (95% CI)	*n*/*N* (%)	Adjusted hazard ratio (95% CI)
**Age (years)**					
18–64	383/743 (52%)	218/743 (29%)	0.95 (0.79, 1.13)	165/525 (31%)	**0.67 (0.55, 0.82)**
65–74	677/974 (70%)	377/974 (39%)	1.02 (0.88, 1.18)	300/597 (50%)	0.93 (0.79, 1.10)
75–84	903/1194 (76%)	495/1194 (41%)	0.95 (0.83, 1.09)	408/699 (58%)	**0.82 (0.71, 0.96)**
85–94*	638/764 (84%)	341/764 (45%)	1	297/423 (70%)	1
**Sex**					
Male*	1331/1746 (76%)	763/1746 (44%)	1	568/983 (58%)	1
Female	1270/1929 (66%)	668/1929 (35%)	0.94 (0.84–1.04)	602/1261 (48%)	**1.14 (1.01–1.28)**
**Charlson comorbidity index**					
0^α^	0	0	NA	0	NA
1–2*	277/713 (39%)	118/713 (17%)	1	159/595 (27%)	1
3–4	392/665 (59%)	181/665 (27%)	**1.35 (1.07, 1.71)**	211/484 (44%)	1.23 (1.00, 1.52)
5+	1932/2297 (84%)	1132/2297 (49%)	**2.59 (2.13, 3.15)**	800/1165 (69%)	**2.09 (1.75, 2.50)**
**Admission type**					
Non-Elective*	2095/2418 (87%)	1168/2418 (48%)	1	927/1250 (74%)	1
Elective	506/1257 (40%)	263/1257 (21%)	**0.58 (0.50–0.68)**	243/994 (24%)	**0.42 (0.36–0.50)**
**Admitted from**					
Home*	1452/2282 (64%)	831/2282 (36%)	1	621/1451 (43%)	1
Hospital	1121/1359 (82%)	588/1359 (43%)	**0.85 (0.77–0.95)**	533/771 (69%)	**0.86 (0.76–0.98)**
Other and unknown	28/34 (82%)	12/34 (35%)	**0.95 (0.54–1.68)**	16/22 (73%)	**1.91 (1.16–3.16)**
**Cancer site**					
Breast (C50)	120/534 (22%)	60/534 (11%)	**0.34 (0.26, 0.45)**	60/474 (13%)	**0.23 (0.17, 0.31)**
Digestive organs (C15–26)	708/867 (82%)	399/867 (46%)	1.11 (0.98, 1.26)	309/468 (66%)	0.98 (0.85, 1.13)
Lung (C30–32, 34, 37, 38)	458/489 (94%)	265/489 (54%)	**1.20 (1.04, 1.39)**	193/224 (86%)	**1.44 (1.22, 1.70)**
Urinary tract (C64–68)	139/314 (44%)	71/314 (23%)	**0.7 (0.55, 0.90)**	68/243 (28%)	**0.6 (0.46, 0.78)**
Other	1176/1471 (80%)	636/1471 (43%)	1	540/835 (65%)	1

Data in bold indicates significant findings. Data are *n* (%) and represent status at index admission to an intermediate care unit. Cancer site (ICD-10 code), full details Additional File Tables C1 and C4. Adjusted hazard ratio (95% CI). ^α^Charlson index cancer is a weighted factor; hence, zero is not possible in the cancer model. ^a^Hazard of death in the first 28 days of admission. ^b^Hazard of death within 29 to 365 days of admission, which excludes patients who died before 28 days. Definition: home—usual place of residence in the community including personal dwelling and care home (with or without nursing)

In the non-cancer model, typically all covariates remained significant overtime compared to the respective reference covariate (Table 2). The model showed a good fit with high concordance at 0.725. Females showed a consistently lower hazard ratio relative to males men (0 to 28 days aHR 0.80 [95% CI, 0.75–0.85]), and elective compared to non-elective admissions (0 to 28 days aHR 0.81 [95% CI 0.73–0.90]). Admissions from hospital showed the highest risk of mortality at 0 to 28 days (aHR 1.26 [95% CI 1.18–1.35]) compared with from usual residence (e.g. home). Hazard ratios increased with increasing comorbidities (Charlson Index) and age and remained consistent overtime. Conversely, the hazard ratios for main diagnosis on index admission were generally highest in the 0 to 28 days post-admission, and decreased overtime. Prominent conditions relative to MSK disorders comprised pneumonia (aHR 6.17 [95% CI 4.90–7.76]), chronic heart disease (adjusted HR 6.14 [95% CI 4.83–7.81]), dementia (aHR 5.07 [95% CI 3.80–6.77]), COPD (aHR 5.01 [95% CI 3.78–6.62]), renal and genitourinary (aHR 3.05 [95% CI 2.41–3.87]) and cerebrovascular disease (aHR 3.02 [95% CI 2.35–3.88]).

The six covariates in the cancer model showed greater variation overtime and slightly lower model concordance of 0.686 compared to the non-cancer model (Table 3). The cancer model showed that patients admitted post-acute had a lower hazard ratio compared with those transferring from usual residence (0 to 28 days aHR 0.85 [95% CI 0.77–0.95] and 29 to 365 days aHR 0.86 [95% CI 0.76–0.98]). Of those admitted from home, 58.9% had a Charlson index of ≥ 3 compared to 40.3% admitted from hospital. Patients with increasing comorbidities (Charlson index ≥ 5) showed the largest ratio (aHR 2.59 [95% CI 2.13–3.15] 0–28 days) relative to the lowest index (1–2). Hazard ratios varied by cancer type with lung cancer showing highest ratio (aHR 1.20 [95% CI 1.04–1.39] 0 to 28 days) relative to ‘other’ cancer conditions. Mortality increased with rising age, but a pattern of higher hazard ratios was less apparent compared with the non-cancer model.

## Discussion

### Main findings

This study reports a novel analysis of the characteristics of a national cohort of adults admitted to intermediate care units and outcome of death over 1 year. The findings show that this is a mainly older population with chronic progressive disease and multimorbidity. Over one in four were in the last year of life. The wide variation in end of life indicates the need for care and treatment to support both recovery and plan for and anticipate end of life. The findings on the associations with end of life identify triggers for palliative care including the main diagnosis (respiratory conditions, dementia, liver disease and cancer), high multimorbidity (≥ 5 Charlson index score), advanced age and non-elective admission. This is a population with high care needs including management of multiple progressive conditions, supporting recovering and anticipating and planning for end of life. The findings challenge perceptions of intermediate care settings, with relatively low technology, as managing seemingly simplistic care needs. New models of care are indicated with better integration between intermediate care facilities, geriatric care and palliative care, and a skilled workforce to manage multiple care needs across the continuum of care and into end of life.

We compared our findings on associations with end of life with 12 studies included in our systematic review on evidence before the study (Additional file D: Table D1). We extracted the relative risk estimates for the factors associated with mortality reported in 11 studies with estimates from our study presented alongside (Additional file D: Table D2). The synthesis of the relative risk data informs factors associated with increasing risk of end of life encompassing demographic, illness and environmental factors. Our study was unique to identify the main diagnosis as a key factor for risk of end of life. These ‘triggers’ against a background of multimorbidity and frailty can precipitate marked functional decline and risk of adverse outcome of, for example, death [4]. However, the review findings demonstrate how environmental factors of quality of care and level of skilled care provision impacted on mortality. Lower regulatory rating of quality of care was associated with higher hazard ratios compared with top quality rating of care (1 star quality rating 1.15 aHR [1.11–1.20] ref. 5 star quality rating) [21]. Similarly, improved survival was associated with a higher staff ratio to number of patients [20] and availability of skilled nurses per bed (e.g. < 4.1 beds per registered nurse post-acute trauma 0.84 aHR [0.77–0.91] and surgery patients 0.80 aHR [0.75–0.86] ref. > 6.7 beds per nurse) [19].

Intermediate care units are an important part of the continuum of services for older people at points of deterioration in health, or when in transition between hospital, home or care home. The provision of palliative care is a commonly cited function of intermediate care units nationally [10] and internationally, but provision varies widely depending on service commissioning and if palliative care is formally recognised in the wider health system [11]. Sezgin et al.’s systematic review identified these settings as delivering key elements of an effective model of care for older people with multimorbidity [8]. Key elements include the presence of a multi-disciplinary team to provide a single-point of entry for multiple interventions from self-management of chronic disease, recovery and rehabilitation, to palliative and end of life care [8]. Although studies report of patient experiences of palliative care in these settings is better compared to hospital, little information is available about the provision or contribution of palliative care advice and support [8]. Our findings show the importance of palliative with over one in four patients in the last year of life. The nearness of death is comparable with acute hospitals (28.8%) [38]. Our findings identify triggers for palliative care assessment including patients with chronic progressive conditions common in old age, such as dementia, respiratory and cardiac conditions, and cancer, high comorbidities (≥ 5 Charlson Index score) and advanced age. The progressive conditions identified are often accompanied by serious health-related suffering likely to benefit from palliative care [1]. However, risk of end of life was also associated with an ‘acute’ stressor event such as pneumonia or injury. The high risk of poor outcome of death for older people with multimorbidity and chronic conditions requires the provision of palliative care alongside treatments supporting recovery. The global COVID-19 pandemic demonstrated the vulnerability of older people to acute decline and death from infection, with highest deaths in older age groups [39].

Our findings identify priority patient groups likely to benefit from care orientated towards quality of life and comprehensive palliative care assessment, and the requirement for delivery by skilled practitioners to ensure high-quality care. The inherent uncertain illness trajectories and wide variation in mortality requires parallel planning to support both recovery and plan for and anticipate end of life. The findings corroborate a model of care across the care continuum with a range of integrated services including geriatric care with intended outcomes emphasising function and recovery, and palliative care with outcomes orientated towards symptoms and concerns and quality of life [40]. Triggers for care are advocated as informed by likelihood of benefit and intended outcomes rather than prognosis when inherently uncertain for this older population group. Using validated comprehensive outcome measures in routine care is demonstrated to improve detection of unmet needs and individual priorities, provision of the right care at the right time and outcomes of care [41], for example, using the Integrated Palliative care Outcome Scale to assess person-centred comprehensive care needs in routine care [42]. New models of care are indicated with integration between intermediate care units, geriatric care, hospice care and palliative care, and for a workforce skilled in geriatric care and palliative care with access to specialists for patients with complex care needs. However, there are challenges for intermediate care facilities. Concerns are reported to ensure levels of competency in for example palliative care and timely equitable access to specialist services based on patient need to support complex clinical decision making on, for example, managing intractable pain [9–11, 43–45]. Young et al. assert that a key area for development in intermediate care facilities is investment in training and support for staff to provide effective care for patients with multiple and complex needs [44].

### Strengths and limitations

A strength of this study is analysis of complete national datasets linked at the individual level. This enabled examination of the total population admitted to intermediate care units in England. The use of incident sampling from index admission date and end of life within 1 year gives original detailed understanding on individual need to inform policy and practice. The findings are relevant for inpatient settings caring for older people, such as Skilled Nursing Facilities in the USA and Care of the Older Person, general medical wards in acute hospitals. Our findings demonstrate individual-level factors as triggers for palliative and end of life care for older people in inpatient facilities. This extends understanding beyond the system-approach reported in prevalence studies using consensus data [38]. Nevertheless, our study has limitations. Our systematic review findings identified the association between mortality and clinical factors (e.g. functional disability) [13, 15, 16, 18, 22], and environmental (e.g. skill mix) [18–21] (Additional file D: Table D2). The use of HSE data impeded wider exploration of associations with clinical variables or intra-variations and heterogeneity between facilities that are common [8, 10, 11, 44]. The cancer group formed a small proportion (4.8%) of the total cohort. Cancer may be under-reported with inclusion limited to the main diagnosis of the index admission, as opposed to a history of cancer. Interpretation of the main diagnosis on the index admission is treated with caution as some diagnostic groups formed a small proportion of the total cohort, for example, patients with liver disease.

## Conclusion

This national study on patients in the last year of life in intermediate care units shows that the traditional emphasis on rehabilitation and recovery has changed with an ageing population and the need for greater integration of palliative care. Adults admitted to these facilities are mainly older with multiple care needs associated with chronic progressive conditions and inherent uncertain illness trajectories of recovery or continued decline. Over one in four patients admitted died within 1 year. Those living with chronic progressive conditions, cancer and multimorbidity had increased risk of end of life. Palliative care must be integrated as part of comprehensive care in intermediate care facilities that encompasses the paramount importance of quality of life for an increasingly older population. Our findings are relevant for acute inpatient hospital facilities caring for older people to align care with the needs of ageing populations. Future research should evaluate new models of care for intermediate care facilities that integrate geriatric care and palliative care across the continuum of care and into end of life, with attention to economic evaluation of investment in workforce training and skill mix to deliver high-quality care.

## Supplementary Information

**Additional file 1: Part A – reporting guideline**. STROBE statement – checklist of items, and RECORD extension for routine data. **Part B – additional methods**. Detailed statistical data analysis plan. **Part C – additional results**. Table C1: Main diagnoses on index admission by cancer and non-cancer. Table C2-C3: Description of the 220 included community hospitals. Table C2: Using individual level patient routine data. Table C3: Using data from the hospital websites. Table C4-C5: Main diagnoses definitions and groupings by ICD-10 codes. Table C4: Main diagnoses identified in the cohort. Table C5: Main diagnoses not identified and identified in the cohort. Fig. C1: Kaplein Meir curve by age categories for cancer and non-cancer groups. **Part D – Evidence before this study on risk of mortality following admission to a sub-acute inpatient community facility**. Methods of review. Fig. D1: PRISMA flow chart of the systematic review process. Table D1: Review of previous studies – study characteristics. Table D2: Factors associated with mortality following admission to a community hospital.

## Data Availability

The data were produced by Public Health England from (1) Hospital Episode Statistics (HES), NHS Digital. Copyright© 2020, re-used with the permission of NHS Digital. All rights reserved, and (2) linked HES-ONS Mortality extract, Office for National Statistics (ONS)© Crown copyright 2020, NHS Digital© Copyright 2020, re-used with the permission of NHS Digital. All rights reserved.

## Abbreviations

aHR: Adjusted hazard ratios
CHA: Community Hospital Association
COPD: Chronic obstructive pulmonary disease
HES: Hospital Episode Statistics
ICD-10: International Classification of Diseases, 10th edition
IQR: Interquartile range
LSOA: Lower Super Output Area
MSK: Musculoskeletal
NHS: National Health Service
ONS: Office for National Statistics
PH: Proportional hazards
