# Supplementary material for: Characteristics and mortality rates among patients requiring intermediate care: a national cohort study using linked databases
Source: BMC Med. 2021 Feb 12;19:48. doi: 10.1186/s12916-021-01912-x (PMC7880511; doi:10.1186/s12916-021-01912-x)
Supplement: Supplementary file 1 — Additional file 1: Part A – reporting guideline. STROBE statement – checklist of items, and RECORD extension for routine data. Part B – additional methods. Detailed statistical data analysis plan. Part C – additional results. Table C1: Main diagnoses on index admission by cancer and non-cancer. Table C2-C3: Description of the 220 included community hospitals. Table C2: Using individual level patient routine data. Table C3: Using data from the hospital websites. Table C4-C5: Main diagnoses definitions and groupings by ICD-10 codes. Table C4: Main diagnoses identified in the cohort. Table C5: Main diagnoses not identified and identified in the cohort. Fig. C1: Kaplein Meir curve by age categories for cancer and non-cancer groups. Part D – Evidence before this study on risk of mortality following admission to a sub-acute inpatient community facility. Methods of review. Fig. D1: PRISMA flow chart of the systematic review process. Table D1: Review of previous studies – study characteristics. Table D2: Factors associated with mortality following admission to a community hospital. [file 12916_2021_1912_MOESM1_ESM.docx]

**Additional files**

**Additional files to**: Evans CJ, Potts L, Dalrymple U, Pring A, Verne J, Higginson IJ, and Wei G. ‘Integrating palliative care in community hospitals: a national cohort study using linked databases’

|  | Pages |
| --- | --- |
| **Part A – reporting guideline** |  |
| STROBE statement – checklist of items, and RECORD extension for routine data | 2-8 |
| **Part B – additional methods** |  |
| Detailed statistical data analysis plan | 9-10 |
| **Part C – additional results** |  |
| Table C1: Main diagnoses on index admission by cancer and non-cancer | 11 |
| Table C2-C3: Description of the 220 included community hospitals |  |
| *Table C2: Using individual level patient routine data* | 12 |
| *Table C3: Using data from the hospital websites* | 13 |
| Table C4-C5: Main diagnoses definitions and groupings by ICD-10 codes |  |
| *Table C4: Main diagnoses identified in the cohort* | 14 |
| *Table C5: Main diagnoses not identified and identified in the cohort* | 15 |
| Figure C1: Kaplein Meir curve by age categories for cancer and non-cancer groups | 16 |
| **Part D – Evidence before this study on risk of mortality following admission to a sub-acute inpatient community facility, including community hospitals, and intermediate care, rehabilitation and skilled nursing facilities** |  |
| Methods of review | 17 |
| Figure D1: PRISMA flow chart of the systematic review process | 18 |
| Table D1: Review of previous studies – study characteristics | 19-24 |
| Table D2: Factors associated with mortality following admission to a community hospital | 25-30 |

**Part A: STROBE statement - checklist of items, and RECORD statement extensions for routine data**

|  | **Item No.** | **STROBE items** | **Location in manuscript item reported** | **RECORD items** | **Location in manuscript item reported** |
| --- | --- | --- | --- | --- | --- |
| **Title and abstract** | | | | | |
|  | 1 | (a) Indicate the study’s design with a commonly used term in the title or the abstract (b) Provide in the abstract an informative and balanced summary of what was done and what was found | Pages 1-2 | RECORD 1.1: The type of data used should be specified in the title or abstract. When possible, the name of the databases used should be included.  RECORD 1.2: If applicable, the geographic region and timeframe within which the study took place should be reported in the title or abstract.  RECORD 1.3: If linkage between databases was conducted for the study, this should be clearly stated in the title or abstract. | Page 2  Page 2  Page 2 |
| **Introduction** | | | | | |
| Background rationale | 2 | Explain the scientific background and rationale for the investigation being reported | Page 3-4 |  |  |
| Objectives | 3 | State specific objectives, including any prespecified hypotheses | Page 4-5 |  |  |
| **Methods** | | | | | |
| Study Design | 4 | Present key elements of study design early in the paper | Page 5 |  |  |
| Setting | 5 | Describe the setting, locations, and relevant dates, including periods of recruitment, exposure, follow-up, and data collection | Page 5 |  |  |
| Participants | 6 | *(a) Cohort study* - Give the eligibility criteria, and the sources and methods of selection of participants. Describe methods of follow-up | Page 5 | RECORD 6.1: The methods of study population selection (such as codes or algorithms used to identify subjects) should be listed in detail. If this is not possible, an explanation should be provided.  RECORD 6.2: Any validation studies of the codes or algorithms used to select the population should be referenced. If validation was conducted for this study and not published elsewhere, detailed methods and results should be provided.  RECORD 6.3: If the study involved linkage of databases, consider use of a flow diagram or other graphical display to demonstrate the data linkage process, including the number of individuals with linked data at each stage. | Pages 5-4  Pages 4-5, HES and APC data dictionaries provide further detail  Page 5, and Figure 1 – flow diagram of the cohort identification and selection |
| Variables | 7 | Clearly define all outcomes, exposures, predictors, potential confounders, and effect modifiers. Give diagnostic criteria, if applicable. | Page 6 | RECORD 7.1: A complete list of codes and algorithms used to classify exposures, outcomes, confounders, and effect modifiers should be provided. If these cannot be reported, an explanation should be provided. | Pages 5-6  Supplemental tables C4 and C5 |
| Data sources/ measurement | 8 | For each variable of interest, give sources of data and details of methods of assessment (measurement).  Describe comparability of assessment methods if there is more than one group | Pages 5-6  Pages 6-7 |  |  |
| Bias | 9 | Describe any efforts to address potential sources of bias | Page 5 and figure 1 details identification of all the community hospitals in England, and the eligibility criteria. We used individual level data over 1-year post-admission, not a census date to minimise bias of longer length of stay. (see pages 4 and 10) |  |  |
| Study size | 10 | Explain how the study size was arrived at | National study, all patients meeting eligibility (page 5) |  |  |
| Quantitative variables | 11 | Explain how quantitative variables were handled in the analyses. If applicable, describe which groupings were chosen, and why | Pages 6-7, and supplemental additional methods |  |  |
| Statistical methods | 12 | (a) Describe all statistical methods, including those used to control for confounding  (b) Describe any methods used to examine subgroups and interactions  (c) Explain how missing data were addressed  (d) *Cohort study* - If applicable, explain how loss to follow-up was addressed  (e) Describe any sensitivity analyses | Pages 6-7, and supplemental additional methods  Pages 6-7, and supplemental additional methods  The multivariate analysis involved complete cases only (pages 6 and 8)  N/A retrospective  Sensitivity analysis in differences in mortality between cancer and non-cancer to inform modelling (page 6). No comparable data in other countries prohibited sensitive analysis of the full modelling procedure. |  |  |
| Data access and cleaning methods |  | . | Page 7 –details data access by respective authors  Pages 5-6, cleaning method | RECORD 12.1: Authors should describe the extent to which the investigators had access to the database population used to create the study population.  RECORD 12.2: Authors should provide information on the data cleaning methods used in the study. | Page 7 –details data access by respective authors  Pages 5-6 |
| Linkage |  | .. | Individual-level linkage, page 5 | RECORD 12.3: State whether the study included person-level, institutional-level, or other data linkage across two or more databases. The methods of linkage and methods of linkage quality evaluation should be provided. | Individual-level linkage, page 4 |
| **Results** | | | | | |
| Participants | 13 | (a) Report the numbers of individuals at each stage of the study (*e.g.*, numbers potentially eligible, examined for eligibility, confirmed eligible, included in the study, completing follow-up, and analysed)  (b) Give reasons for non-participation at each stage.  (c) Consider use of a flow diagram | Pages 7-8, and figure 1  N/A retrospective data  See Figure 1 | RECORD 13.1: Describe in detail the selection of the persons included in the study (*i.e.,* study population selection) including filtering based on data quality, data availability and linkage. The selection of included persons can be described in the text and/or by means of the study flow diagram. | Pages 7-8, and figure 1 |
| Descriptive data | 14 | (a) Give characteristics of study participants (*e.g.*, demographic, clinical, social) and information on exposures and potential confounders  (b) Indicate the number of participants with missing data for each variable of interest  (c) *Cohort study* - summarise follow-up time (*e.g.*, average and total amount) | Page 7, and table 1  Complete case only multivariate analysis, page 8  1-year follow-up from index admission date |  |  |
| Outcome data | 15 | *Cohort study* - Report numbers of outcome events or summary measures over time | Figure 2, A-C, and appendix figure C1 Tables 1, 2 and 3, and pages 7-9 |  |  |
| Main results | 16 | (a) Give unadjusted estimates and, if applicable, confounder-adjusted estimates and their precision (e.g., 95% confidence interval). Make clear which confounders were adjusted for and why they were included  (b) Report category boundaries when continuous variables were categorized (c) If relevant, consider translating estimates of relative risk into absolute risk for a meaningful time period | Tables 2-3, pages 7-9  Tables 2-3 e.g. age  NA |  |  |
| Other analyses | 17 | Report other analyses done—e.g., analyses of subgroups and interactions, and sensitivity analyses | Multivariate models of cancer and non cancer groups, page 8 |  |  |
| **Discussion** | | | | | |
| Key results | 18 | Summarise key results with reference to study objectives | Page 9 |  |  |
| Limitations | 19 | Discuss limitations of the study, taking into account sources of potential bias or imprecision. Discuss both direction and magnitude of any potential bias | Pages 11 | RECORD 19.1: Discuss the implications of using data that were not created or collected to answer the specific research question(s). Include discussion of misclassification bias, unmeasured confounding, missing data, and changing eligibility over time, as they pertain to the study being reported. | Pages 11-12 |
| Interpretation | 20 | Give a cautious overall interpretation of results considering objectives, limitations, multiplicity of analyses, results from similar studies, and other relevant evidence | Page 13 |  |  |
| Generalisability | 21 | Discuss the generalisability (external validity) of the study results | Page 13 |  |  |
| **Other Information** | | | | | |
| Funding | 22 | Give the source of funding and the role of the funders for the present study and, if applicable, for the original study on which the present article is based | Pages 7 and 12 |  |  |
| Accessibility of protocol, raw data, and programming code |  | .. |  | RECORD 22.1: Authors should provide information on how to access any supplemental information such as the study protocol, raw data, or programming code. | Appendix part B, additional methods, and author correspondence details. |

**Part B: Detailed statistical analysis plan**

For all analyses, patients were followed up from the index admission date for 12 months. All analyses were completed in R version 3·6·1. The cohort formed two groups comprising non-cancer conditions or cancer identified from ICD-10 codes on primary diagnosis on the index admission. Descriptive statistics were used to report demographic data for the total cohort and by the respective sub-group, and the hospital characteristics. Kaplan Meier curves using the observed data were used to display survival probabilities by the respective sub-groups and key prognostic factors.

Survival analysis used two multivariate models of complete cases (whereby the selected predictive independent covariates were not missing) by the respective sub-group termed as the cancer model or non-cancer model. Note, patients in the non-cancer model may have had a secondary diagnosis of cancer, but their admission was for a non-cancer condition. The data was assembled in an appropriate format for survival analysis whereby each person admitted to a community hospital in 2016 either died within a year of admission, or were “censored” if they survived. Information on age, gender, ethnicity, primary reason for admission, comorbidities, admission location, admission type, number of hospital wards, level of deprivation formed covariates.

Both models were Cox proportional hazards (PH) models with time dependent coefficients [36, 46], the time taken for an “event” to occur (here defined as a patient’s death) was modelled. The risk of dying at any given time t is given by the hazard function, h(t) where:

$$h\left( t \right)=h_{0}\left( t \right) \times exp(\beta_{1}(t)x_{1}+\ldots+\beta_{p}(t)x_{p})$$

Where $h_{0}$ represents a baseline hazard function, and there are $p$ covariates (denoted by $x$) with time dependent coefficients $\beta_{1}\ldots\beta_{p}$.

Time dependent coefficients were used to account for non-proportional hazards whereby the coefficients associated with covariates change during the follow-up period. In this model, higher values of $h\left( t \right)$ equated to a higher probability of dying at time $t$. The values of $exp(\beta_{i})(t)$ were hazard ratios associated with each covariate, where a hazard ratio with a value higher than one increases $h\left( t \right)$, and a hazard ratio with a value lower than one decreases $h\left( t \right)$. We used a stepwise variable selection procedure to obtain the best Cox’s proportional hazards model model, using the ‘My.stepwise.coxph’ function from the ‘My.stepwise’ package in R [47]. This method uses bidirectional selection with an entry criteria of 0·1 and stay criteria of 0·05. The final models were selected considering models that contained the fewest covariates with the maximum concordance statistic, and variables of clinical importance (e.g. categories of cancer) with presentation to inform clinical interpretation (e.g. grouping age categories).

These selected covariates were then assessed for violation of the proportional hazards assumption. This assumption is violated when the effect of a covariate on survival changes over time. For such variables, it is inappropriate to fit a single linear coefficient. A Cox proportional hazard model was fitted with the base covariates, and the ‘cox.zph’ function from the ‘survival’ package in R [47] was implemented to test the proportional hazards assumption for each base covariate. Each variable with a two-sided p-value of less than 0·05 was considered to have non-proportional hazards and a time dependent term was added into the final Cox proportional hazard model for each.

Both the cancer and non-cancer models initially violated the proportional hazards assumption. To address this coefficient could vary over time as step functions, i.e. by fitting different coefficient values for different time intervals. The chosen interval boundaries were 28 days in the cancer model and 28 days and 180 days in the non-cancer model. This means that the dataset was split into time dependent parts and modelled using the same covariates within the relevant model for ease of comparison. This was undertaken using the ‘survSplit’ function from the ‘survival’ package in R [48] interval boundaries were selected by visualising the departure of proportionality by plotting the average hazard estimate of a given covariate and the time varying hazard of the same covariate over time. This resulted in:

$$Cancer h\left( t \right)= \left\{ \begin{aligned} h_{0,1}\left( t_{1} \right) \times\exp\left( \beta_{1,1}{\left( t_{1} \right)x}_{1}+\ldots+\beta_{p,1}{\left( t_{1} \right)x}_{p} \right)where t_{1}\leq28 days \\ h_{0,2}\left( t_{2} \right) \times\exp\left( \beta_{1,2}\left( t_{2} \right)x_{1}+\ldots+\beta_{p,2}{\left( t_{2} \right)x}_{p} \right) where 28<t_{2} \leq365 days \end{aligned} \right.$$

$$Non-Cancer h\left( t \right)= \left\{ \begin{aligned} h_{0,1}\left( t_{1} \right) \times\exp\left( \beta_{1,1}\left( t_{1} \right)x_{1}+\ldots+\beta_{p,1}{\left( t_{1} \right)x}_{p} \right) where t_{1}\leq28 days \\ h_{0,2}\left( t_{2} \right) \times\exp\left( \beta_{1,2}{\left( t_{2} \right)x}_{1}+\ldots+\beta_{p,2}{\left( t_{2} \right)x}_{p} \right) where 28<t_{2} \leq180 days \\ h_{0,3}\left( t_{3} \right) \times\exp\left( \beta_{1,3}{\left( t_{3} \right)x}_{1}+\ldots+\beta_{p,3}\left( t_{3} \right)x_{p} \right) where 180<t_{3} \leq365 days \end{aligned} \right.$$

With adjustments, the models met the proportional hazards assumption and the hazard ratios for covariates showed variation overtime. The respective model fit was assessed by visually comparing observed to expected hazard plots and observing optimal concordance statistics. We reported adjusted hazard ratios and 95% confidence intervals.

**Part C – additional results**

**Table C1: Main diagnosis on index admission by cancer and non-cancer**

|  | **Main diagnosis on index admission** | **n (%)** |
| --- | --- | --- |
| **Cancer site** | | 3680 (4·8%) |
|  | Digestive organs | 870 (23·6%) |
|  | Breast | 535 (14·5%) |
|  | Respiratory and intrathoracic organs (lung) | 489 (13·3%) |
|  | Urinary tract | 315 (8·6%) |
| ‘Other’ (grouped in the cancer model) | |  |
|  | Malignant neoplasm ill-defined, secondary and unspecified sites | 494 (13·4%) |
|  | Lymphoid, haematopoietic and related tissue | 266 (7·2%) |
|  | Male genital organs | 261 (7·1%) |
|  | Female genital organs | 146 (4·0%) |
|  | Eye, brain and other parts of central nervous system | 121 (3·3%) |
|  | Mesothelial and soft tissue | 73 (2·0%) |
|  | Lip, oral cavity and pharynx | 35 (1·0%) |
|  | Melanoma and other malignant neoplasms of skin | 35 (1·0%) |
|  | Independent (primary) multiple sites | 18 (0·5%) |
|  | Bone and articular cartilage | 14 (0·4%) |
|  | Thyroid and other endocrine glands | 8 (0·2%) |
| **Non-cancer conditions** | |  |
|  | Injury | 13 006 (17·0%) |
|  | Musculoskeletal disorders | 9231 (12·0%) |
|  | Renal and genitourinary diseases | 4827 (6·3%) |
|  | Stroke | 3067 (4·0%) |
|  | Digestive diseases | 2828 (3·7%) |
|  | Pneumonia | 2822 (3·7%) |
|  | Mental and behavioural disorders | 2433 (3·2%) |
|  | Chronic heart disease | 1822 (2·4%) |
|  | Infections | 1411 (1·8%) |
|  | Chronic Obstructive Pulmonary Disease | 1126 (1·5%) |
|  | Dementia | 1081 (1·4%) |
|  | Acute heart disease | 955 (1·3%) |
|  | Blood diseases | 575 (0·8%) |
|  | Liver disease | 178 (0·2%) |
| ‘Other’ (grouped in the non-cancer model), ‘other’ main categories included: | | 27 662 (36·1%) |
|  | R69 Unknown and unspecified causes of morbidity | 9730 (12·7%) |
|  | ICD chapter R (excluding R69) | 7780 (10·1%) |
|  | Other ICD chapters (excluding R) | 10 152 (13·2%) |

**Table C2: Description of the 220 included community hospitals using patient data**

|  | **Non-Cancer cases** | **Cancer cases** | **All cases** |
| --- | --- | --- | --- |
| **Number of patients** | 73 024 | 3680 | 76 704 |
| **Length of stay community hospital** | |  |  |
| 0 nights | 4724 (6·5%) | 192 (5·2%) | 4916 (6·4%) |
| 1-7 nights | 17 289 (23·8%) | 1538 (41·9%) | 18 827 (24·7%) |
| 8-21 nights | 20 378 (28·1%) | 1059 (28·8%) | 21 437 (28·1%) |
| 22-90 nights | 28 004 (38·6%) | 852 (23·2%) | 28 856 (37·8%) |
| 91+ nights | 2254 (3·1%) | 32 (0·9%) | 2286 (3·0%) |
| **IMD quintile (based on patients postcode)** | | | |
| I (most deprived) | 9944 (1·7%) | 395 (10·8%) | 10 339 (13·6%) |
| 2 | 15 258 (21·0%) | 809 (22·0%) | 16 067 (21·1%) |
| 3 | 17 407 (24·0%) | 1020 (27·8%) | 1427 (24·2%) |
| 4 | 16 251 (22·4%) | 854 (23·3%) | 17 105 (22·4%) |
| 5 (least deprived) | 13 698 (18·9%) | 592 (16·1%) | 14 290 (18·8%) |
| **Rural/Urban locality of the hospital** | | | |
| 1 (Urban) | 1283 (2%) | 71 (2%) | 1354 (2%) |
| 2 | 1678 (2%) | 150 (4%) | 1828 (2%) |
| 3 | 1522 (2%) | 139 (4%) | 1661 (2%) |
| 4 | 806 (1%) | 86 (2%) | 892 (1%) |
| 5 | 45 529 (62%) | 1945 (53%) | 47 474 (62%) |
| 6 | 11 065 (15%) | 624 (17%) | 11 689 (15%) |
| 7 | 7629 (10%) | 468 (13%) | 8097 (11%) |
| 8 Rural | 3187 (4%) | 192 (5%) | 3379 (4%) |
| Missing postcode | 326 (0%) | 5 (0%) | 331 (0%) |
| **Region in England hospital located** | | | |
| Cheshire and Merseyside | 2926 (4%) | 186 (5%) | 3112 (4%) |
| East Midlands | 9446 (13%) | 349 (9%) | 9795 (13%) |
| East of England | 4040 (6%) | 430 (12%) | 4470 (6%) |
| Greater Manchester, Lancashire and South Cumbria | 6694 (9%) | 247 (7%) | 6941 (9%) |
| London | 2058 (3%) | 50 (1%) | 108 (3%) |
| Northern England | 5173 (7%) | 360 (10%) | 5533 (7%) |
| South East Coast | 5718 (8%) | 108 (3%) | 5826 (8%) |
| South West | 14 964 (20%) | 788 (21%) | 15 752 (21%) |
| Thames Valley | 3325 (5%) | 35 (1%) | 3360 (4%) |
| Wessex | 6979 (10%) | 514 (14%) | 7493 (10%) |
| West Midlands | 6774 (9%) | 290 (8%) | 7064 (9%) |
| Yorkshire and The Humber | 4760 (7%) | 314 (9%) | 5074 (7%) |
| Unknown | 168 (0%) | 9 (0%) | 177 (0%) |
| Abbreviation: IMD - index of multiple deprivation indice[2] quintiles based on Lower Super Output Area (LSOA) derived from patients’ residential postcodes identified from HES data records. | | | |

**Table C3: Summary of the hospital services as reported on hospital websites**

|  | **Non-Cancer** | **Cancer** | **All** |
| --- | --- | --- | --- |
| General medical care | 101 (37·0%) | 78 (38·6%) | 102 (36·8%) |
| Rehabilitation | 55 (20·1%) | 35 (17·3%) | 57 (20·6%) |
| Rehabilitation and palliative care | 52 (19·0%) | 42 (20·8%) | 52 (18·8%) |
| Rehabilitation and stroke rehabilitation | 9 (3·3%) | 5 (2·5%) | 9 (3·2%) |
| Rehabilitation, stroke rehabilitation and palliative care | 2 (0·7%) | 1 (0·5%) | 2 (0·7%) |
| Rehabilitation, palliative care and, children and adolescent mental health services | 1 (0·4%) | 1 (0·5%) | 1 (0·4%) |
| General medical, rehabilitation and stroke rehabilitation | 13 (4·8%) | 10 (5·0%) | 13 (4·7%) |
| General medical care and palliative care | 2 (0·7%) | 2 (1·0%) | 2 (0·7%) |
| Geriatric care, rehabilitation and palliative care | 3 (1·1%) | 3 (1·5%) | 3 (1·1%) |
| Geriatric care | 3 (1·1%) | 3 (1·5%) | 3 (1·1%) |
| Geriatric care and rehabilitation | 3 (1·1%) | 1 (0·5%) | 3 (1·1%) |
| Intermediate care | 2 (0·7%) | 1 (0·5%) | 2 (0·7%) |
| Orthopaedic rehabilitation, palliative care and stroke rehabilitation | 1 (0·4%) | 1 (0·5%) | 1 (0·4%) |
| General medical care, mental health and rehabilitation | 1 (0·4%) | 1 (0·5%) | 1 (0·4%) |
| Geriatric mental health and rehabilitation | 3 (1·1%) | 2 (1·0%) | 3 (1·1%) |
| Dementia care, mental health and rehabilitation | 1 (0·4%) | 1 (0·5%) | 1 (0·4%) |
| Dementia care, psychiatric rehabilitation, rehabilitation, stroke rehabilitation | 1 (0·4%) | 1 (0·5%) | 1 (0·4%) |
| Mental health, neuro-rehabilitation and rehabilitation | 2 (0·7%) | 1 (0·5%) | 2 (0·7%) |
| Acute mental health | 1 (0·4%) | 1 (0·5%) | 1 (0·4%) |
| Unknown | 17 (6·2%) | 12 (5·9%) | 18 (6·5%) |
| **Total** | 273 | 202 | 277 |

**Table C4: Main diagnoses identified in the cohort: defined by ICD-10**

| **Cancer site (ICD-10 codes)** | |
| --- | --- |
|  | Digestive organs (C15-26) |
|  | Respiratory and intrathoracic organs (C30-32, C34, C37, C38) |
|  | Breast (C50) |
|  | Urinary tract (C64-C68) |
|  | ‘Other’ (in the cancer model) |
|  | Malignant neoplasms of ill-defined, secondary and unspecified sites (C76-C80) |
|  | Lymphoid, haematopoietic and related tissue (C81-C85, C88, C90-C95), |
|  | Male genital organs (C60, C61, C62) |
|  | Female genital organs (C51, C52, C53, C54, C55, C56, C57) |
|  | Eye, brain and other parts of central nervous system (C69-C72) |
|  | Mesothelial and soft tissue (C45, C48, C49) |
|  | Lip, oral cavity and pharynx (C00-C11, C13, C14) |
|  | Melanoma and other malignant neoplasms of skin (C43, C45) |
|  | Independent (primary) multiple sites (C97) |
|  | Bone and articular cartilage (C40-41) |
|  | Thyroid and other endocrine glands (C73-C75) |
| **Non-cancer conditions (ICD-10 codes)** | |
|  | Injury (S00-S06, S10- S16, S19-S22, S24, S27, S29-S32, S34, S36, S37, S39, S40-S44, S46, S48-S53, S56, S58-S63, S66, S68-S73, S75, S76, S78-S83, S86, S88-S93, S96-S99, T00-T02, T08-T17, T20-, T21, T22-T25, T29, T30, T67, T68, T74, T75, T78-T88) |
|  | Musculoskeletal disorders (M00, M05-M08, M10-M25, M31-M35, M40, M41, M43, M45-M51, M53, M54, M60, M62, M65, M66, M67, M70-M72, M75, M76, M77, M79-M81, M83-M90, M92- M96, M99) |
|  | Genitourinary diseases (N02, N04 N05, N10-N15, N17-N21, N23, N25, N28, N30-N32, N34-N36, N39-N43, N45, N47-N50, N60-N64, N71-N73, N76, N80-N90, N92, N93, N94, N95, N99) |
|  | Cerebrovascular disease (I60-68) |
|  | Digestive diseases (K00-K02, K04-K08, K10-K14, K20-K22, K25-K31, K35, K37, K40- K46, K50-K52, K55- K66, K80-K83, K85, K86, K90-K92) |
|  | Pneumonia (J12-J15, J17, J18) |
|  | Mental and behavioural disorders (F05, F06, F07, F09-F15, F17, F19, F20-F25, F28-F34, F39-F45, F50, F51, F53, F59-F62, F64, F69, F70, F79, F81, F84, F90, F99) |
|  | Chronic heart disease (I05, I07-I11, I13, I31, I34-I36, I38, I42, I50, I70, I95) |
|  | Infections (A01, A04, A05, A08, A09, A15, A17, A19, A32, A31, A39, A40, A41, A46, A48, A49, A69, A80, A81, A86, A87, B00, B02, B07, B25, B27, B30, B34-B37, B44, B49, B50, B76, B86, B94, B95, B96, B99) |
|  | Chronic obstructive pulmonary disease (J40-J44) |
|  | Dementia (F00-03, G30-31) |
|  | Acute heart disease (I20, I21, I24, I25, I146-I149, I51) |
|  | Blood diseases (D50, D51, D52, D53, D56, D58-D65, D68-D70, D73, D75, D76, D80, D86, D89) |
|  | Liver disease (B17-B18, C22, K70-B75) |
| **Abbreviation**: ICD-10 International Classification for Diseases - 2010 | |

###

**Table C5: All diagnoses both not identified and identified in the cohort: defined by ICD-10**

| **Cancer site (ICD-10 codes)** | |
| --- | --- |
|  | Digestive organs (C15-26) |
|  | Respiratory and intrathoracic organs (C30-34, C37-C39) |
|  | Breast (C50) |
|  | Urinary tract (C64-C68) |
|  | ‘Other’ (in the cancer model) |
|  | Malignant neoplasms of ill-defined, secondary and unspecified sites (C76-C80) |
|  | Lymphoid, haematopoietic and related tissue (C81-C89, C90-C96), |
|  | Male genital organs (C60, C61, C62, C63) |
|  | Female genital organs (C51, C52, C53, C54, C55, C56, C57, C58) |
|  | Eye, brain and other parts of central nervous system (C69-C72) |
|  | Mesothelial and soft tissue (C45, C46, C47, C48, C49) |
|  | Lip, oral cavity and pharynx (C00-C11, C13, C12, C14) |
|  | Melanoma and other malignant neoplasms of skin (C43, C45) |
|  | Independent (primary) multiple sites (C97) |
|  | Bone and articular cartilage (C40-41) |
|  | Thyroid and other endocrine glands (C73-C75) |
| **Non-cancer conditions (ICD-10 codes)** | |
|  | Injury (S00-S08, S10-S73, S75, S76, S78-S99, T00-T21, T22-T35, T66-T88) |
|  | Musculoskeletal disorders (M00-02, M03-M9) |
|  | Genitourinary diseases (N00, N03, N06-N09, N16, N22, N24, N26, N27, N29, N33, N37, N38, N44, N46, N51-N59, N65-N70, N74, N75, N77-N79, N89, N91, N96-N98) |
|  | Cerebrovascular disease (I60-68) |
|  | Digestive diseases (K00- K69, K78-K99) |
|  | Pneumonia (J12-J18) |
|  | Mental and behavioural disorders (F05, F06, F07, F09-F15, F17, F19, F20-F25, F28-F34, F39-F45, F50, F51, F53, F59-F62, F64, F69, F70, F79, F81, F84, F90, F99) |
|  | Chronic heart disease (I05-I11, I13, I31, I34-I36, I38, I42, I50, I70, I95) |
|  | Infections (A00-A99, B00-B14, B20-B96, B99) |
|  | Chronic obstructive pulmonary disease (J40-J44) |
|  | Dementia (F00-04, G30-31) |
|  | Acute heart disease (I2 -I25, I39 -I41, I146-I149, I51) |
|  | Blood diseases (D50- D89) |
|  | Liver disease (B15-B19, C22, K70-K75, K77) |
| **Abbreviation**: ICD-10 International Classification for Diseases - 2010 | |

**Figure C1:** **Kaplein-Meir curve by age categories for cancer and non-cancer groups**

###
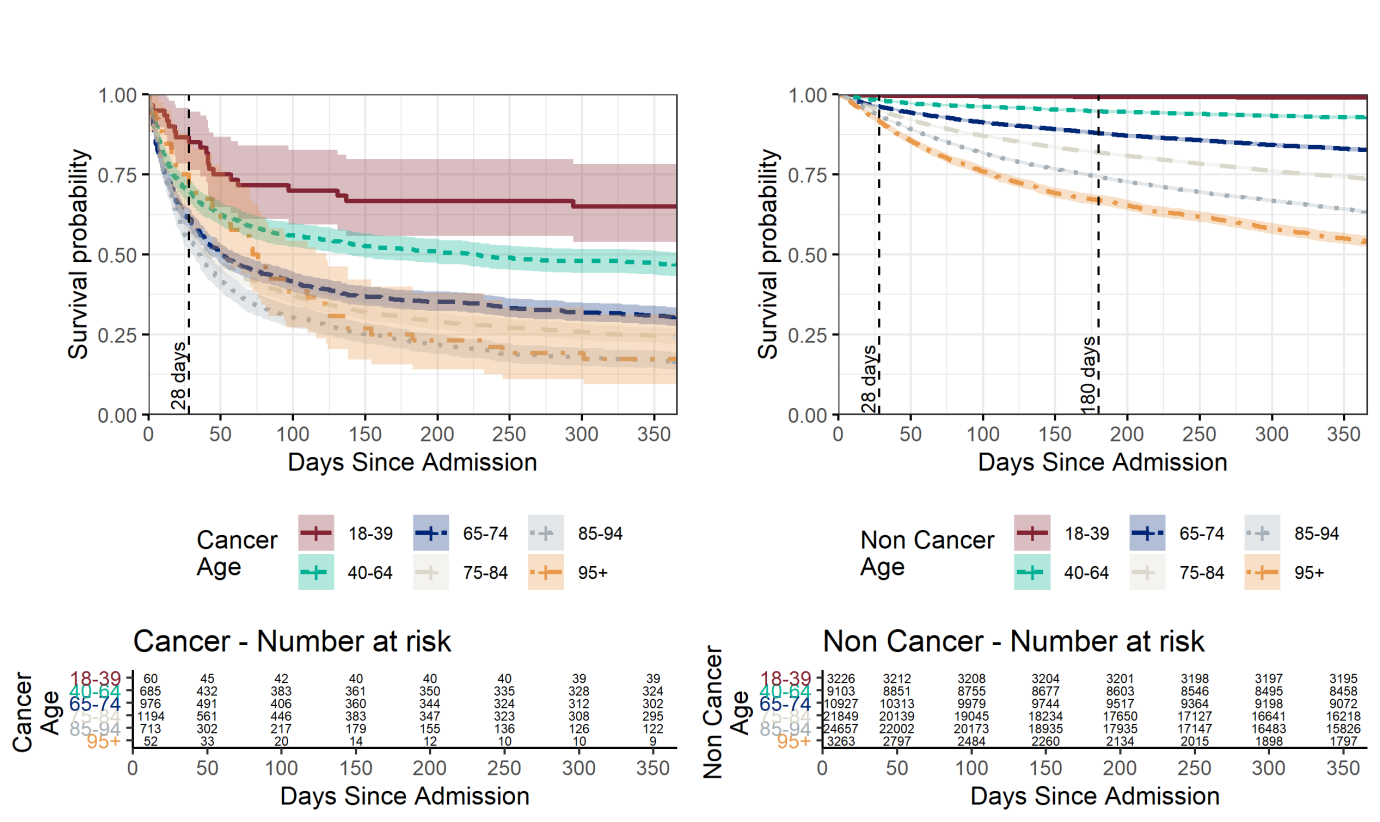


**Part D – Evidence before this study on risk of end-of-life following admission to an intermediate care facility defined as sub-acute inpatient facilities including community hospitals, intermediate care units, post-acute, rehabilitation and skilled nursing facilities**

**Methods**

**Search strategy:** We searched PubMed and OVID MEDLINE for observational studies published in English from Jan 1, 2009, to Dec 31, 2019 using search terms for mortality and community hospitals (e.g. skilled nursing facilities [SNFs]) We used MeSH terms “Intermediate Care Facilities” OR “Skilled Nursing Facilities” OR “Hospitals, community” AND “Mortality” OR “Survival analysis”, limited to English language and year 2009 to 2019. Key words “community hospital$”.

**Inclusion criteria:**  Study setting – an intermediate care unit defined as a service model that: 1) provides a range of non-acute services to a local community, 2) is led by community-based health professionals; and 3) provides inpatient beds. Services broadly encompass post-acute care, rehabilitation, intermediate care, care of the elderly, and/or palliative care. Population adults - aged 18 years or over. Outcomes - include mortality or survival. Study design - observational.

**Exclusion criteria:**  Study setting: community-based hospitals providing acute services and treatments (for example district general hospital); Intermediate care units (e.g. high dependency unit) as a step-down from ICU and step-up from ward level care; maternity services only; paediatric services only; outpatient services only; or long-term care facility (e.g. nursing home).

**Extraction of study characteristics and results**: Data were extracted from each eligible study including author, country of origin, year, study design, aim, setting/data source, population, age profile and relative risk estimates for the factors associated with the outcome mortality/survival

**Data analysis**: Descriptive characteristics of the eligible studies are presented in table form, and the outcome data is displayed and collated reporting on the prevalence of mortality, and the associated factors. Identification and collation of results/discussion on ‘need for palliative or end of life care’.

**Results**

Figure D1 details the PRISMA flow diagram reporting study screening and identification. Twelve studies met eligibility reporting ~661 751 participants (range 18 to 414 997) from the USA (n=7), Asia Pacific region (n=3), and Europe (n=2). Most reported age (n=10) and involved patients aged ≥ 65 years (average range 66.0 to 85.7 years) (n=9) (see table D1). Facilities provided post-acute care for the elderly in SNFs (n=6), or general elderly care and rehabilitation (n=3). 19.3% of patients died on average within the year (range 10% to 47.8%, 7 studies). We examined the relative risk estimates for mortality to identify significant factors for end of life (see table D2). Factors included: 1) demographics of increasing age, and male sex; 2) illness characteristics of increasing comorbidities, and dependency; and 3) environment of the community hospital (e.g less nursing hours per patient day) and discharge location (e.g. acute hospital). No studies considered the total population admitted to a community hospital, or the imminence of end of life and the need for palliative care.

**Figure D1: PRISMA flow chart of the systematic review process**

Records identified through database searching total N=753

(MEDLINE n=268; PubMed n= 485)

## Identification

Duplicates removed
(n = 262)

Records screened at title/abstract
(n = 491)

Records excluded
(n =465)

## Screening

**Full-text articles excluded, with reasons (n = 14)**

1. Setting: long-term care setting (n=1); or acute care community hospital (n=8)
2. Population: community-dwelling (n=1)
3. Outcome: discharge setting (n=2); acute hospital readmission (n= 1); length of stay (n=1)

**Eligibility**

Full-text articles assessed for eligibility
(n = 26)

Reference chaining n =5

Number of studies included in the synthesis (n=12)

**Included**

**Table D1: Review of previous studies – study characteristics**

| **Author, yr, country** | **Aim** | **Data source/ setting** | **Population** | **Age profile** | **Mortality** | **Adjusted for covariates** | **Study size** | **Average follow-up** |
| --- | --- | --- | --- | --- | --- | --- | --- | --- |
| Chen LK,  2010 [12]  Taiwan | To evaluate the effectiveness of a community-hospital based post-acute care programme (CH-PAC) vs. reference group discharge to nursing home. | Two community-hospitals affiliated with the discharging acute 2hospital | Patients with functional decline after acute hospital admission | CH-PAC mean age 85.7 years (SD 5.1)  Reference group mean age 85.4 years (SD 5.3) | Mortality 15.6% CH-PAC group vs. 23.9% reference group at 1-year | Model 2: age, gender, acute hospital LoS, pre-discharge  BI | n=96 CH-PAC group  vs.  n=234 reference group | 1-year |
| Chen C, 2014 [13] Singapore | To identify predictors of mortality and to describe the combined effect of comorbidity and disability on mortality for patients admitted for inpatient rehabilitation from acute hospital. | National hospital database extracted medical records for all rehabilitation community hospitals (n=4) | Patients admitted 1996-2005 for rehab from acute disability (e.g. hip fracture, stroke). | Mean age 73.0 years (SD 29.0) at index admission | Annual mortality median 855.5 (10.0%) range 473 (5.5%) to 1046 (12.2%)  66.9% (n=8 565) over 10.9 years | Age, year admitted, sex, main diagnosis | N=12 804 patients in the final cohort | Median 10.9 years |
| Dahl, 2015 [14] Norway | To investigate the effectiveness of hospital discharges to a municipality with an intermediate community hospital (ICH) compared to municipality without ICH | Patient reported at index stay, follow-up on hospital use and mortality from Norwegian Patient Register, general hospital and university hospital’s respective registers. | Post-acute care for patients aged 60 years and over discharged from an acute hospital and requiring at least 3 days in-patient care, and expected to return home after ICU admission. | Mean age 78.3 years (SD 8.6) | 15.5% (n=17)  (excluded patients with short-life expectancy, lacked capacity to give consent) | Age, sex, number of  diagnoses, functional status and having primary health  care (yes/no) at the index stay | n=110 ICH  (n=99 ICH-home care; n=119 municipality no ICH) | 1-year |
| Ehlenbach 2019 [15], USA | To quantify the cognitive and physical impairment for survivors of severe sepsis discharged to a skilled nursing facility [SNF] and relationship between impairment and mortality | Medicare patient data 2005-2009, linked with Minimum Data Set-Cognition scale (MDS-COGS) and activities daily living (MDC-ADL) | Patients surviving severe sepsis discharged from acute hospital to SNF. Random 5% sample | Mean age 82.1 years (SD 7.7) at discharge | Discharge to SNF 1-year mortality 47.8% (n=31 834)  Newly discharged SNF (n=31 114) 42.2%, survival median 19.4 mnths; Discharged back to SNF (n=35 426), 52.8 %, survival median 10.4 mnths | Age, sex, race, rurality, Medicaid eligibility, comorbidities (Elixhauser index), and during acute hospitalisation received mechanical ventilation, or admitted ICU | N=66 540 | 1-year  Also report median survival analysis to death |
| Greenough 2014 [23], USA | To examine mortality for patients requiring long-term care chronic ventilation discharged to SNF or remain Long-term care chronic vent unit (LTCV) | Patient medical records Johns Hopkins Medical Centre and transfers to SNFs Baltimore, MD, region | Chronically ill patients dependent on long-term mechanical ventilation assessed as stable with less complex care needs | Not stated | 1-year mortality 70% transfer to SNF; vs. 15% remain LTCV Kaplein-Meier Survival Log rank *P*-value =0.251(no CIs) | No multivariate analysis | N=18  Transferred SNF n=7  Remain LTCV n=11 | 66 months |
| Hakkarainen 2016 [16] USA | To evaluate risk factors for failure to return home and death for older patients discharged to a SNF, and develop a predictive tool for likelihood of adverse outcomes | Medicare data linked with MDS data and National Death Index for SNFs between Jan 2007 to Dec 2009 in 5 states. | Older patients hospitalized for non-trauma surgical, trauma or stroke diagnoses and discharged to SNFs for post-acute care | Mean age 78.5 years (SD 10.7) | 1-year mortality 26.1%  3-year mortality 31.6%.  30% deaths occurred in a SNF | Age, sex, Charlson Comorbidity Index, admission priority, LoS, ICU stay, ADL score, cognitive status, parenteral nutrition pressure ulcers at admission to SNF, blood transfusion in acute hospital | N= 414 997 | 1-year factors associated mortality |
| Myint 2012, [17] UK | To examine if FANGG and AIMs are predictors of mortality for hospitalised older people, when morbid Rankin function score is considered | Medical records of patients admitted to a community hospital or acute hospital care of the elderly wards, April to August 2004, and April to August 2005 | Consecutive admissions to care of the elderly wards and a community hospital | Mean age 85.3 years, range 64-104 years | 18% (n=72) in hospital deaths | Age, sex, FANGG, AIMS x Rankin interaction, hospital site. | N=400  Community hospital n=266  Acute hospital n=134 | Until discharge, death or 90 days |
| Nehra 2016, [18] USA | To describe the characteristics of trauma patients admitted to an Inpatient Rehabilitation Facility [IPF] and outcomes including death within 1-year compared matched cohort no IRF. | Washington State trauma rehabilitation registry for the 14 IRF units in the state, linked with the Comprehensive Hospital Abstract Reporting System database between 2011 and 2012 | Patients discharged to an IRF, require 1) intensive  rehabilitation;2) therapy in at least 2 modalities; and 3) supervision by  rehabilitation physician. | Mean age 50.5 years (SD 23.5) | Mortality prevalence not reported.  78.2% patients discharged home | Not reported | n=993 IRF n=631 matched cohort no IRF | 1-year factors associated mortality |
| Thornblade 2018, [19] USA | To determine if SNF-level factors are associated with hospital readmission, failure to discharge home and death. | Medicare-certified SNFs, a non-random sample of beneficiaries from 5 states between 2007-2009 | Post-acute care for adult patients hospitalised for trauma or major surgery discharged to a SNF | Mean age 78 years | Mortality 23% for trauma patients and 26% for surgery patients | Age, sex, CCI, ADL, ICU stay, parenteral nutrition, pressure ulcers, cognitive status, emergency admission; and for trauma patients injury severity | N=389 133  (3707 SNFs) | 1-year |
| Tong 2011, [20] USA | To investigate how regulatory change in staff levels in SNFs affects nurse employment and patient mortality | Annual SNF data from the California Office of Statewide Health Planning and Development, 1995-2002 | Patients requiring 24-hour low-acuity medical care. | Not reported | Post-law 2000-2002. Low staffing  average deaths 36.4 (SE 28.4) (1836 observations); High staffing average 40.4 (SE 44.6) (600 observations) | Patient acuity by including SNF fixed effects, fixed trends, and SNF level patient demographics | 2436 observations post-law 2000-2002; 4060 observations pre-law 1995-1999 (612 SNFs) | Deaths during SNF admission |
| Unroe 2012, [21] USA | To examine the associations between quality ratings of SNFs and readmission, and mortality for patients with heart failure | Medicare Provider and Analysis Review claims data for patients discharged from hospital to SNF, between 2006-2007 | Primary diagnosis of heart failure, admitted to a SNF within 2 days of hospital discharge | Median age 84 years (IQR 76-89) | 28.5% (n=46 950) | Model 1: age, sex, race, comorbidity, Medicaid eligibility, LoS index admission > 7 days, hospitalized, rural, region, and year admitted. Model 2: number of beds, type of ownership, located hospital, part of chain | N=164 672  [admitted to 13 619 SNFs] | 90 days |
| Venkataraman 2016, [22] Singapore | To identify factors associated with functional gain, discharge setting, and survival after admission to a rehabilitation community hospital | Medical record review for 4 community hospitals between Jan, 1996 to Dec, 2005 | Lower leg amputation and diabetes requiring post-acute rehab | Mean age 66.0 years (SD 10.8) | 1-year mortality 11% (n=26/235)  Median survival 69 months after discharge | Hospital and year of admission | N=256 | Survival censor date 31 Dec 2011 |
| Evans 2020,  UK | To analyse the factors associated with mortality following intermediate care unit admission to inform the need for palliative care | Linked individual-level National Health Service Hospital Episode Statistics and Office for National Statistics death registration data | Adults (≥ 18 years) admitted for one or more nights to an eligible intermediate care unit between 01/01/2016 to 31/12/2016, excluding out patients and maternity. | 76·7 years (SD=15·0) | 1-year mortality 28.0% | Time dependent coefficients for explanatory variables which had a non-stationary effect on mortality overtime (e.g. main diagnosis) | N=76 704 | 1-year from index admission date |
| Abbreviations: ADL activities of daily living; AIMs acute illness markers; CH-PAC community-hospital based post-acute care programme; CCI Charlson comorbidity index; FANGG fracture, acquired neurological deficit or any geriatric giant; ICH intermediate community hospital; ICU Intensive care unit; IPF inpatient rehabilitation facility; SNF skilled nursing facility; Long-term care chronic vent unit (LTCV) | | | | | | | | |

**Table D2: Factors associated with end of life post-admission to an intermediate care facility reported in multivariate analysis/economic modelling**

|  | **Demographic factors** | | | | | | | | | | | **Illness factors** | | | | | | **Environment** | |
| --- | --- | --- | --- | --- | --- | --- | --- | --- | --- | --- | --- | --- | --- | --- | --- | --- | --- | --- | --- |
| Author, yr, aHR/aOR | Sub-groups | Age in years | Sex | Marital status | | Ethnicity | | Caregiver available | | Socio economic status | | Main diagnosis | Co-morbidities | Physical disability | | Cognitive disability | | CH facilities | Admission/ discharge / LoS |
| Chen LK 2010 [12]  aHR  n=96 CH-PAC; n=234 nursing home | x | | x | x | | x | | x | | x | | x | x | x | | x | | CH-PAC programme mortality, Model 2, 0.38 (0.19 – 0.76) vs. nursing home | |
| Chen C 2014 [13]  aHR  N=12 804 | x | | x | Widowed 1.47(1.34-1.61) or married 1.19(1.08-1.30) ref single | | x | | Caregiver available 1.19 (1.10-1.28) ref no carer | | Low SES 1.40 (1.29-1.53) ref high SES | | x | Charlson index high (≥ 7) and BI all disability 4.42 (3.89-5.03) vs. ref either alone | | | x | | CH: B 1.10(1.03-1.22), D 1.21(1.05-1.38) ref A | Discharge to NH 1.37 (1.28-1.47), or acute hospital 1.96 (1.84-2.09) ref home |
|  |  |  |  |  |  |  |  |  |  |  |  |  | Comorbidities (≥ 7) 2.80 (2.49-3.16) ref none | Total disability 2.44 (2.28-2.61) ref no/ mild | |  |  |  |  |
| Dahl 2015 [14]  aHR  n=110 ICH, n=119 no ICH | x | | x | x | | x | | x | | x | | x | x | x | | x | | Mortality aHRs admitted ICH, compared region no ICH. No significant difference detected | |
| Ehlenbach 2019 [15]  aHR  N=66 540 | x | | x | x | | x | | x | | x | | x | x | Barthel index limited to total disability 1.2 - 4.3 vs. ref independent | | Moderate to very severe 1.4 - 3.1 ref intact | | x | x |
| Hakkarainen 2016 [16]  aHR  N= 414 997 | **Trauma** | 65to≥85, 1.33 - 1.77 ref ≤64yrs | Male 1.85 (1.30-1.33) | x | | x | | x | | x | | Pressure ulcers  1.57 (1.52-1.64) | Charlson Index  1 to ≥3, 1.65-3.31 ref none | MDS-ADL  Scores 4-5, 2.08-4.44 ref none | | Modified to severe 1.61-3.71 ref none | | x | Readmitted acute hospital HR 28.2 (27.2-29.3) ref not readmitted [excluded predictive model] |
|  | **Surgical** | 65to≥85, 1.12- 1.37 ref ≤64yrs | Male 1.31 (1.30-1.33) | x | | x | | x | | x | | 1.65(1.62-1.69) | 1 to ≥3, 1.57-2.91 ref none | Scores 2-6, 1.32-3.59 ref none | | Modified to severe 1.44-2.64 ref none | | x |  |
|  | **Stroke** | 65to≥85, 1.50-2.91 ref≤64yrs | Male 1.34 (1.26-1.42) | x | | x | | x | | x | | 1.40(1.33-1.48) | 2 to ≥3, 1.43-2.07 ref none | Sores 3-6, 1.83-4.80 ref none | | Modified to severe 1.28-3.39 ref none | | x |  |
| Myint 2012 [17]  aHR N=400 | x | | Male 1.99 (1.09-3.63) | x | | x | | x | | x | | x | FANGG and AIMs none sign. | Rankin score 5, 3.31 (1.48-7.40) ref score 0-4 | | x | | CH vs. acute hospital, none sig. | x |
| Nehra 2016 [18]  aHR, n=993 IRF, n=631 matched cohort no IRF | 1.05 (1.04-1.07) | | x | x | | x | | x | | x | | Trauma penetrating  3.97 (1.33-11.86) ref blunt | x | At discharge severe dependence (FIM 3-7) 2.84 (1.63-4.95) ref mild dependence (FIM 11-12) | | Rehab 0.60 (0.39-0.92) ref no rehab | | Length of stay 1.03 (1.02-1.03) | x |
| Thornblade 2011 [19]  aOR  N=389 133 | x | | x | x | | x | | x | | x | | Higher surgical density (> 14.1%) 0.89 (0.83-0.96) | x | x | | | | **<4.1 beds per nurse**, trauma 0.84 (0.77-0.91); surgery 0.80 (0.75-0.86) ref >6.7 beds per nurse. **<12 beds per RN**, trauma 0.77 (0.71-83); surgery 0.80 (0.75-0.86) ref >49 beds per RN. **Ratio <1.6 LPNs per RN**, trauma 0.82 (0.76-0.88); surgery 0.78 (0.73-0.84). | |
| Tong 2011 [20]  Instrumental variable [IV]  4896 observations, 612 SNFs. | x | | x | x | | x | | x | | x | | x | x | x | | x | | Low staffing SNF, one unit increase in hours per resident day, 6.14 IV decline deaths during stay, decrease, 1.78 per facility per year, 4.6% decrease from pre-law staffing levels | |
| Unroe 2012 [21]  aHR  N=164 672 | x | | x | x | | x | | x | | x | | x | x | x | | x | | **Quality**:1 star 1.15 (1.11-1.20), 2 star 1.09 (1.04-1.13), 3 star 1.05 (1.01-1.09)) ref 5 star. **Number of beds** Q2 1.05 (1.01-1.09), Q3 1.05 (1.02-1.09), Q4 1.07 (1.03-01.10) ref Q1. **Owner:** State 1.08 (1.03-1.15), Non-profit 0.94 (0.92-0.97), multi-facility owner 1.03 (1.01-1.05) ref for-profit | |
| Venkataraman 2016 [22] aHR N=256 (n=235 in model) | None significant | | None significant | None significant | | Malay (7%) 0.37 (0.16-0.87) ref Chinese (83.6%) | | None significant | | x | | IHD 1.27 (1.27-4.00) ref none; | Charlson index 1 0.43 (0.24-0.77) ref 4+ | Modified BI admission higher function 0.98(0.97-0.99) | | **x** | | Discharge ‘other’ 1.82 (1.02-3.23) ref home. | |
| **Reported study**  Evans 2020  aHR  N=76 704  (n=76 514 complete cases in model) | **Cancer**, 0-28 days | Not sign. | Not sign. | | x | | Excluded in model | | x | | Excluded in model | Lung 1.2 (1.04-1.39) ref ‘other’ cancer | Charlson Index, 5+ 2.59 (2.13-3.15) ref 1-2 | | x | | x | x | Elective admission  0·58 (0·50-0·68) ref non-elective |
|  | 29-365 days | ≤64yrs 0.67(0.55-0.82) ref 85-94+ | Female 1.14 (1.01-1.28) | | x | | Excluded in model | | x | | Excluded in model | Lung 1.44 (1.22-1.70) ref ‘other’ cancer | 5+ 2.09 (1.75-2.50) ref 1-2 | | x | | x | x | 0·42  (0·36-0·50) |
|  | **Non-cancer**  0 -28 days | 0.38 (≤64yrs) to 0.7 (75-84yrs) ref 85-94+ | Female 0·8 (0·75-0·85) | | x | | Excluded in model | | x | | Excluded in model | COPD 5·01 (3·78-6·62);  Pneumonia 6·17 (4·90-7·76); CHD 6·14 (4·83-7·81);  Dementia 5·07 (3·80-6·77)ref MSK | 5^+^ 2·57 (2·36, 2·79) ref 1-2 | | x | | x | x | 0·81  (0·73-0·90) |
|  | 29-180 days | 0.17 (≤64yrs) to 0.6 (75-84yrs) ref 85-94+ | Female 0·76 (0·73-0·80) | | x | | Excluded in model | | x | | Excluded in model | COPD 2·86 (2·45-3·34)  Pneumonia 2·31 (2·03-2·62); CHD 2·41 (2·10-2·76);  Dementia 2·17 (1·82, 2·57)ref MSK | 5^+^ 2·31 (2·19, 2·44) ref 1-2 | | x | | x | x | 0·72  (0·67- 0·77) |
|  | 181- 365 days | 0.14(≤64yrs) to 0.62 (75-84yrs) ref 85-94+ | Female 0·76 (0·72-0·80) | | x | | Excluded in model | | x | | Excluded in model | COPD 2·76 (2·26-3·39);  Pneumonia 2·34 (2·00, 2·74);  CHD 1·89 (1·57-2·28);  Dementia 2·23 (1·80-2·76) ref MSK | 5^+^ 2·06 (1·92, 2·22) ref 1-2 | | x | | x | x | 0·67  (0·62- 0·73) |
| Cox proportional adjusted hazard ratio [aHR] or adjusted odds ratio (95% confidence intervals) for factors showing significant difference compared with reference group, reported by respective category or aHRs/aORs range across scale categories if all show difference at level of significance. N=400^a^ (n=266 community hospital; n=134 acute hospital care of the elderly wards). Abbreviations: BI Barthel Index; CH community hospital; CH-PAC community hospital post-acute care programme; CHD Chronic heart disease; COPD chronic obstructive pulmonary disease; FIM Functional Independence Measure (physical, psychological, social); ICH intermediate community hospital; ICU intensive care unit; IHD Ischaemic heart disease; LoS length of stay; LPN licensed practical nurse (completed approx.1 year of nursing education ); None sign. No significant difference detected; Ref reference group; SES socio economic status; Q1-4 – quartile of certified number of beds [ Q1 (3-70 beds), Q2 (71-102 beds), Q3 (103-132), and Q4 (133-1389)]. | | | | | | | | | | | | | | | | | | | |
